# Mindfulness meditation increases default mode, salience, and central executive network connectivity

**DOI:** 10.1038/s41598-022-17325-6

**Published:** 2022-08-02

**Authors:** Benno Bremer, Qiong Wu, María Guadalupe Mora Álvarez, Britta Karen Hölzel, Maximilian Wilhelm, Elena Hell, Ebru Ecem Tavacioglu, Alyssa Torske, Kathrin Koch

**Affiliations:** 1 Department of Neuroradiology, School of Medicine, Klinikum Rechts Der Isar, Technical University of Munich, Ismaninger Str. 22, 81675 Munich, Germany; 2 TUM-Neuroimaging Center (TUM-NIC), Technical University of Munich, Munich, Germany; 3 Institute of Medical Psychology, Ludwig-Maximilians-Universität München, Munich, Germany; 4 Heidelberg University Hospital, Heidelberg, Germany; 5 Department of Psychology, Ludwig-Maximilians-Universität München, Munich, Germany; 6 Graduate School of Systemic Neurosciences, Ludwig-Maximilians-Universität München, Martinsried, Germany

**Keywords:** Cognitive neuroscience, Psychology

## Abstract

Recent research has begun to identify the neural mechanisms underlying the beneficial impact of mindfulness meditation training (MMT) on health and cognition. However, little is known about the effects of MMT on the global interplay of large-scale networks (LSNs) in the brain. In the present study, healthy, meditation-naïve adults (*N* = 46) underwent resting state fMRI prior to and upon completing 31 days of MMT or an active control intervention. Independent component analysis, sliding time window, and seed-based correlation analyses were performed to assess training-related changes in functional connectivity (FC) within and between networks with relevance to mindfulness meditation. Across sliding time window analyses and seed-based correlation analyses, we found increased FC between nodes of the default mode network (DMN) and nodes of the salience network (SN) in participants of the MMT. Seed-based correlation analyses revealed further connectivity increases between the SN and key regions of the central executive network (CEN). These results indicate, that, among multiple LSNs, one month of mindfulness meditation effectively increases interconnectivity between networks of the triple network model (DMN, SN, CEN), hereby introducing a potential mechanistic concept underlying the beneficial impact of MMT.

**Clinical trial registration:** This study is listed as a clinical trial on the ISRCTN registry with trial ID ISRCTN95197731 (date of first registration: 15/02/2022).

## Introduction

Mindfulness encompasses an individual’s ability or tendency to consciously engage in a state of non-judgmental, present-moment attendance^1^. It is cultivated through the practice of mindfulness meditation which entails a broad range of techniques that typically employ self-induced states of focused attention or open awareness towards sensations or experiences^2^. A continuously growing body of research has revealed that regularly engaging in mindfulness practice can exert beneficial effects on mental and physical health as well as on cognitive capacities such as sustained attention, working memory, and other executive functions^3–6^. In fact, widespread consensus has been reached about its effectiveness in stress-reduction^7,8^, improvement of depression symptomatology^9^, pain-management^10,11^, and health-related quality of life^12^. It has further been successfully implemented in the clinical treatment of various psychiatric conditions, such as anxiety disorders^13^, addictive disorders^9^, and post-traumatic stress disorder^14^.

Thus, in the face of rising demands for mental health care^15,16^, mindfulness meditation might provide a customizable, easy to use, and remotely practicable method to increase mental resilience and overall quality of life. In order to optimize its application, vigorous efforts have been made to untangle the mechanisms which mediate the benefits of mindfulness meditation. Conceptually, mindfulness meditation is believed to primarily act over three axes: Attention control, emotion regulation, and self-awareness, all of which are cognitive qualities which can be enhanced through regular practice^17^. Changes in these domains were found to go along with extensive functional and structural alterations in the brain: the most consistent observations were made in the anterior cingulate cortex (ACC), prefrontal cortex (PFC), posterior cingulate cortex (PCC), insula, and subcortical structures such as the amygdala and the striatum^17^. The interplay of these structures allowed for initial insights into possible neural pathways underlying mindfulness meditation: while the ACC and the PFC are known to play a fundamental role in conflict monitoring and other attentional processes^18,19^, these brain areas have also been found to be more active after mindfulness meditation practice^20^ or in experienced meditators^21^. Repeated findings attribute these regions as having a mindfulness-mediated modulatory impact on activations of the amygdala^22–24^, where responses to emotional stimuli diminished after mindfulness-based interventions^25–27^. Expressions of self-awareness, such as mind-wandering and self-referential processing, have been mostly ascribed to the PCC and medial PFC, which are areas that form key regions of the default mode network (DMN)^28–31^. Abnormalities in DMN function have been associated with a variety of neuropsychiatric disorders^32^, particularly rumination, which is a core symptom of depressive disorders, was found to increase as a function of DMN activation^33^. These key regions of the DMN were observed to be relatively less active in experienced meditators, indicating a potential pillar of the anti-depressant effects of mindfulness meditation^34^. Deactivations within the DMN were accompanied by stronger coupling of these areas with the ACC and PFC, suggesting a mechanism over which attentional control is established over a diverted mind^34^.

Nevertheless, as previous studies are often considerably affected by initial hypotheses and have additionally been conducted under methodologically diverse conditions, the ability to synthesize these reports into a reasonable mechanistic account of mindfulness meditation remains difficult. The simultaneous study of distributed brain areas operating in large-scale networks (LSN) provides an integrative and comprehensive solution to this problem. Within this field, the triple network model has become a paradigm. According to this, neural activity is essentially organized into three LSNs: While the central executive network (CEN) is thought to gain control under task-positive directed conditions, the DMN serves as the task-negative counterpart^35^. In turn, the salience network (SN) evaluates sensory input for its reactive demand and, upon this, modulates engagement of the other two networks^36^. Alterations in the functioning of the triple network model have further been linked to a broad range of neuropsychiatric disorders^35^. While traditional research has been targeting key regions of these networks individually, relatively novel data-driven techniques such as independent component analysis (ICA) make it possible to discretely detect these and other LSNs in functional magnetic resonance imaging (fMRI). It was only recently that such approaches received more attention within the field of mindfulness meditation research. Cross-sectional studies have started to explore the relationship between mindfulness and LSNs by correlating functional connectivity (FC) within and between these networks with common measures of self-reported mindfulness. Findings tentatively suggest that, in individuals with higher levels of mindfulness, areas associated with attentional processes are more connected within several LSNs^37,38^. Others indicate that these individuals display less connectivity between subnetworks of the DMN and between the DMN and the SN^39^. To date, only few studies have addressed the impact of mindfulness meditation training (MMT) on these networks. In one of these, the effects of mindfulness-based stress reduction (MBSR), a standardized 8-week MMT curriculum, on FC were primarily seen in auditory and visual networks^40^. Another study observed decreasing FC within an anterior subnetwork of the DMN in opioid-dependent patients after undergoing a mindfulness-based therapy procedure^41^ while in sixth-grade students anticorrelation between the DMN and CEN increased following 8 weeks of mindfulness training^42^. This gives further grounds to conclude that mindfulness meditation influences brain dynamics in a way that favors attention-related structures over those involved in undirected and inattentive states.

While this shows that LSNs provide a system of operators that are susceptible to the effects of mindfulness meditation, a paucity of coherent information about the leverage that it has on the complex interplay of LSNs remains, and existing inferences are often compromised by methodological constraints. Participant samples are often small, restricted to clinical populations, or have only been observed under pre-defined functional conditions. A large proportion of studies implemented cross-sectional designs and, if intervention-based, often lacked active control conditions. Hence, the present study seeks to investigate the effects of MMT on brain function under functionally independent and methodologically rigorous conditions. A representative sample of clinically healthy participants was pseudo-randomly assigned to 31 days of web based MMT or to an active control condition. Instead of pursuing a priori defined regions of interest (ROIs), we employed data-driven methods to automatically detect intrinsic connectivity networks (ICNs). The selection of networks was then made upon functional–anatomic correspondence to the triple network model and areas that were considered central venues of MMT-induced enhancement of attention control, emotion regulation, and self-awareness (PCC, ACC, insula, PFC, subcortical regions)^17^. In addition to the DMN, SN, and CEN, this included frontal networks, subcortical networks and, as MMT puts strong emphasis on body-awareness, sensorimotor networks. We deem the resulting array of networks to be representative for brain regions susceptible to the effects of MMT and, under the given study conditions, to provide a solid foundation on which to measure the objective impact of mindfulness meditation on brain function.

## Results

In this study, participants were pseudo-randomly allocated to 31 days of a professionally designed web based MMT or a health training (HT) which provided general health-related information. Before and after the intervention, participants underwent magnetic resonance imaging (MRI) and questionnaire-based assessment of subjective levels of mindfulness. To assess MMT-related changes of resting state FC, independent component analysis (ICA), sliding time window analysis, and seed-based correlation analysis were performed and results were compared between time points and groups.

### Sample characteristics

Across groups, no statistically significant differences in age, gender, education (as measured by years spent with school, higher education or professional training), or lifestyle characteristics were detected (Table 1). On average, participants spent 40.6 days (*SD* 6.4) between two scans and completed 29.5 sessions (*SD* 1.8). Scanning intervals and average number of sessions completed were not found to differ significantly between groups. No harms or unintended effects were observed during the course of the study.

**Table 1:** Participant demographics, lifestyle characteristics and scanning intervals.

	Total (*N* = 46)	MMT (*n* = 20)	HT (*n* = 26)	*p* value
**Demographic characteristics**				
Age, M ± SD	35.1 ± 10.4	33.6 ± 8.7	36.3 ± 11.6	0.39
Female, *n* (%)	23 (50)	9 (45)	14 (54)	0.55
Years of education, M ± SD	18.9 ± 5.8	18.1 ± 3.5	19.6 ± 7.0	0.38
**Lifestyle characteristics**				
BMI, (kg/m^2^), M ± SD	24.1 ± 4.2	23.5 ± 3.4	24.6 ± 4.7	0.38
Cigarette smokers, *n* (%)	4 (9)	3 (15)	1 (3)	0.30
Alcohol, M ± SD^†^	2.3 ± 2.5	3.2 ± 3.1	1.7 ± 1.7	0.08
Physical exercise, M ± SD^‡^	173.0 ± 149.5	204.8 ± 156.0	149.4 ± 142.8	0.23
**Days between**				
First scan—second scan, M ± SD	40.6 ± 6.4	40.3 ± 5.1	40.8 ± 7.3	0.80
First scan—first training, M ± SD	1.5 ± 2.4	1.6 ± 1.9	1.4 ± 2.7	0.80
Last training—second scan, M ± SD	8.1 ± 6.5	7.7 ± 4.9	8.4 ± 7.6	0.74
Average number of sessions completed	29.5 ± 1.8	29.5 ± 1.5	29.4 ± 2.0	0.88

^†^Serving sizes of wine (150 ml) or beer (350 ml) per week. ^‡^Minutes per week.

*MMT* Mindfulness Meditation Training, *HT* Health Training, *BMI* Body Mass Index.

### Self-reported mindfulness

A Shapiro–Wilk test did not assess any violations of normality assumptions. On average, MAAS scores slightly decreased across the course of the intervention in participants of the MMT (*M*_*Pre*_ = 62.1, *SD* = 12.8; *M*_*Post*_ = 61.3, *SD* = 8.6), whereas participants of the HT reported higher scores compared to baseline (*M*_*Pre*_ = 62.7, *SD* = 14.3; *M*_*Post*_ = 65.8, *SD* = 11.9).

Results of the ANOVA were found to not be statistically significant (*F* (1,43) = 1.53, *p* = 0.221, partial *η*^2^ = 0.035). To distinguish between conclusive and inconclusive evidence, an additional Bayesian ANOVA was performed, which revealed a *BF*_*10*_ of 0.53 or respectively a *BF*_*01*_ of 1.87 for the group by time interaction, indicating inconclusive evidence.

### Independent component analysis

**Figure 1:**
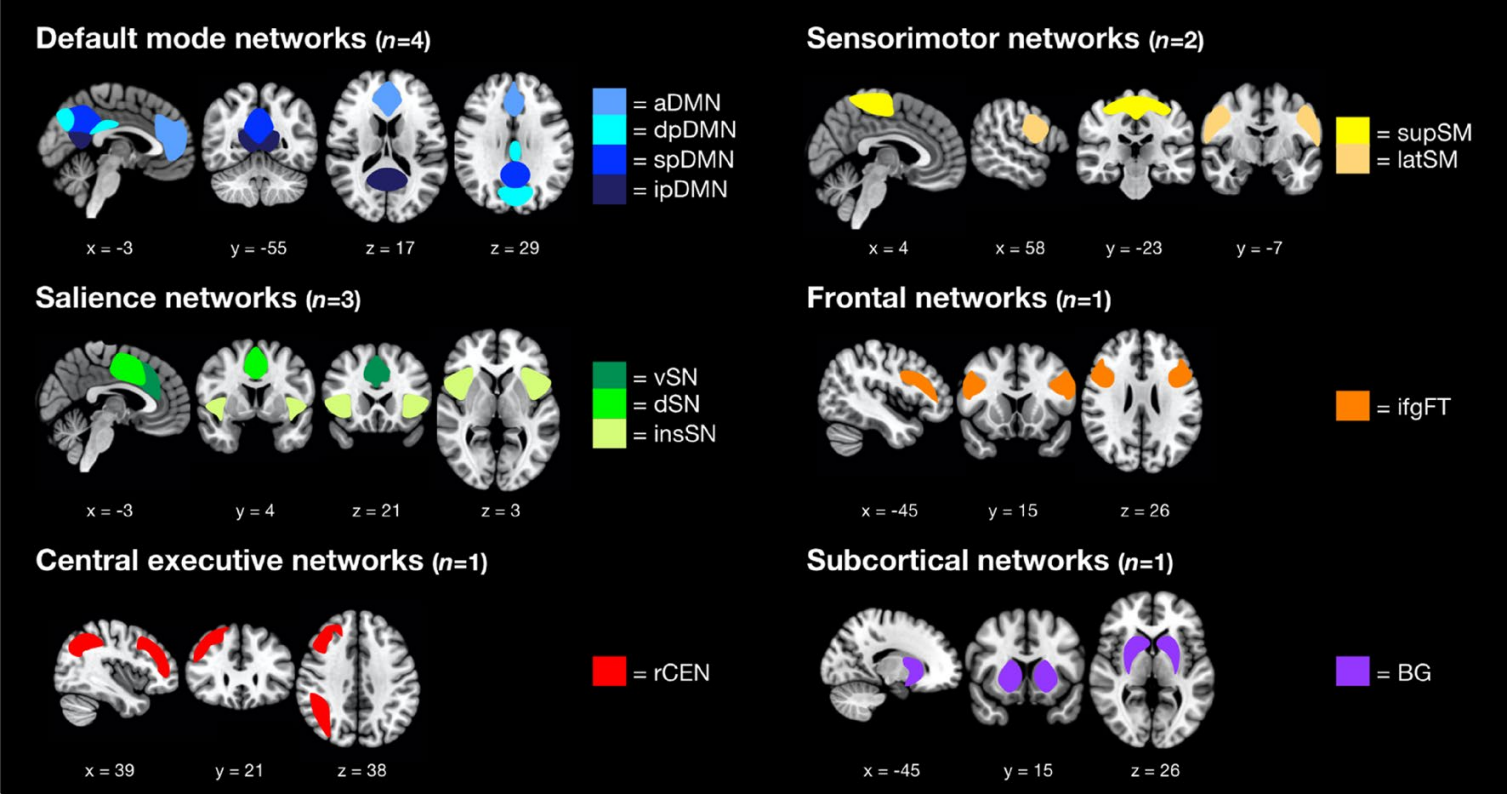
Composite maps of the resulting 12 network components sorted into 6 large-scale networks as displayed on a Montreal Neurological Institute template taken from ICBM 152 Nonlinear atlases version 2009 (© 1993–2004 Louis Collins, McConnell Brain Imaging Centre, Montreal Neurological Institute, McGill University)^43^ (schematic display). Visualized with help of MRIcroGL v1.2.20210317 (https://www.nitrc.org/projects/mricrogl)^44^. *aDMN* anterior default mode network, *dpDMN* dorsal posterior default mode network, *spDMN* superior posterior default mode network, *ipDMN* inferior posterior default mode network, *vSN* ventral salience network, *dSN* dorsal salience network, *insSN* insular salience network, *rCEN* right central executive network, *supSM* superior sensorimotor network, *latSM* lateral sensorimotor network, *ifgFT* inferior frontal gyrus network, *BG* basal ganglia network.

An ICA was performed to extract 12 network components (in the following also called “subnetworks”), subsumed into 6 large-scale networks, which were included for further analysis. (Fig. 1; Supplement Table S1).

#### Static functional connectivity

Analysis of static FC did not detect any statistically significant impact of either training procedure on connectivity within or between networks.

#### Dynamic functional connectivity

**Figure 2:**
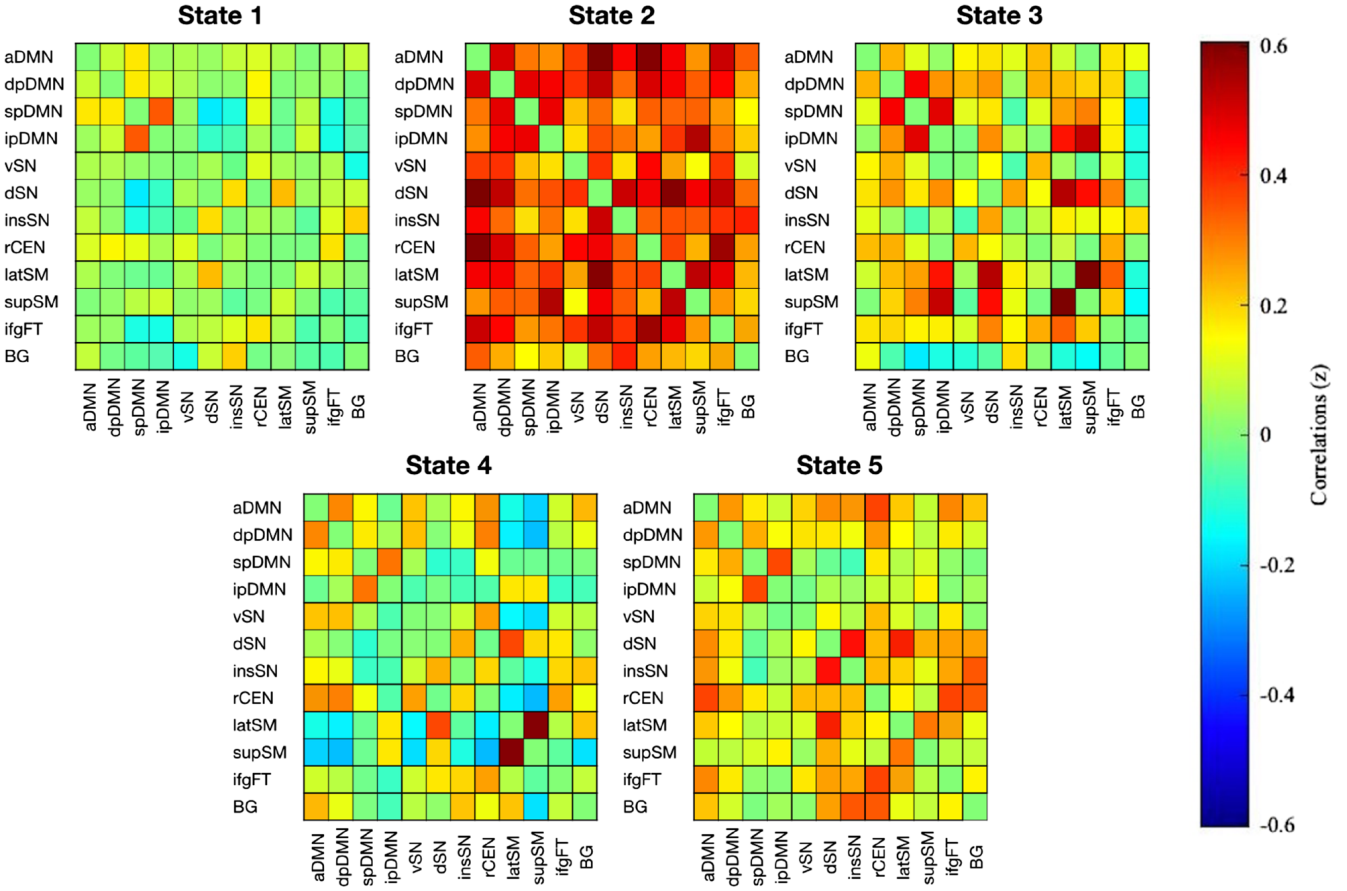
Cluster centroids representing different states of connectivity between network components across both groups and time points. Colors indicate z-transformed connectivity values with warm colors indicating high values and cool colors indicating low values. *aDMN* anterior default mode network, *dpDMN* dorsal posterior default mode network, *spDMN* superior posterior default mode network, *ipDMN* inferior posterior default mode network, *vSN* ventral salience network, *dSN* dorsal salience network, *insSN* insular salience network, *rCEN* right central executive network, *supSM* superior sensorimotor network, *latSM* lateral sensorimotor network, *ifgFT* inferior frontal gyrus network, *BG* basal ganglia network.

Sliding time window analyses allow for the assessment of dynamic FC and revealed five stable and reproducible connectivity states across all participants which are to be roughly characterized in the following (Fig. 2): The first state is generally determined by hypoconnectivity, except for positive FC between two components of the DMN. In contrast, the second state is defined by strong positive correlations between all networks. States three to five all show moderate to low connectivity, distinguishable, however, through anticorrelation of basal ganglia activity with most other networks in state three and anticorrelation of sensorimotor networks with most other networks in state four.

At baseline, none of the features assessed were found to differ significantly across groups. However, after the intervention, mindfulness meditators showed increased connectivity between a subnetwork of the DMN (spDMN) and two subnetworks of the SN (dSN, insSN) within state five (Table 2). No significant changes of interconnectivity were observed within the control group. Yet, participants who completed the HT demonstrated a higher number of occurring states (*M*_*Pre*_ = 19.80, *M*_*Post*_ = *28.64, t* = 3.80, *p* < 0.001) as compared to before the training. This was not the case for participants of the MMT (*M*_*Pre*_ = 24.30, *M*_*Post*_ = *24.55, t* = 0.08, *p* = 0.93). Mean dwell time within states was not found to be affected by any of the courses.

**Table 2:** Fisher r-to-z transformed correlation values of spDMN and dSN as well as spDMN and insSN within state five before and after the intervention for both groups and results of paired t-tests, p value corrected at false-discovery-rate (FDR).

Network pair	MMT				HT			
	Mean z-score		*t* value	*p*_FDR_	Mean z-score		*t* value	*p*_FDR_
	Pre	Post			Pre	Post		
spDMN—dSN	− 0.1665	− 0.0085	5.14	< 0.001	0.0273	0.0012	− 1.16	0.28
spDMN—insSN	− 0.1747	0.0136	5.13	< 0.001	− 0.0106	− 0.0267	− 0.24	0.81

*MMT* Mindfulness Meditation Training, *HT* Health Training, *spDMN* superior posterior default mode network, *dSN* dorsal salience network, *insSN* insular salience network.

**Figure 3:**
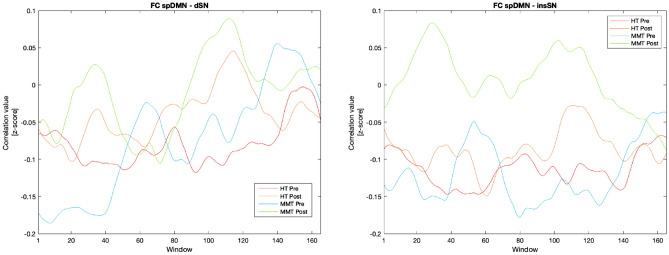
Group level Fisher r-to-z transformed correlation values between superior posterior default mode network (spDMN) and dorsal salience network (dSN) and between spDMN and insular salience network (insSN) across sliding windows. *HT* Health Training, *MMT* Mindfulness Meditation Training.

Figure 3 shows window-wise correlation values between the time course of network pairs plotted along the sliding time window thus illustrating the occurrence of connectivity increments in the MMT group beyond a specific state.

### Seed-based connectivity

**Figure 4:**
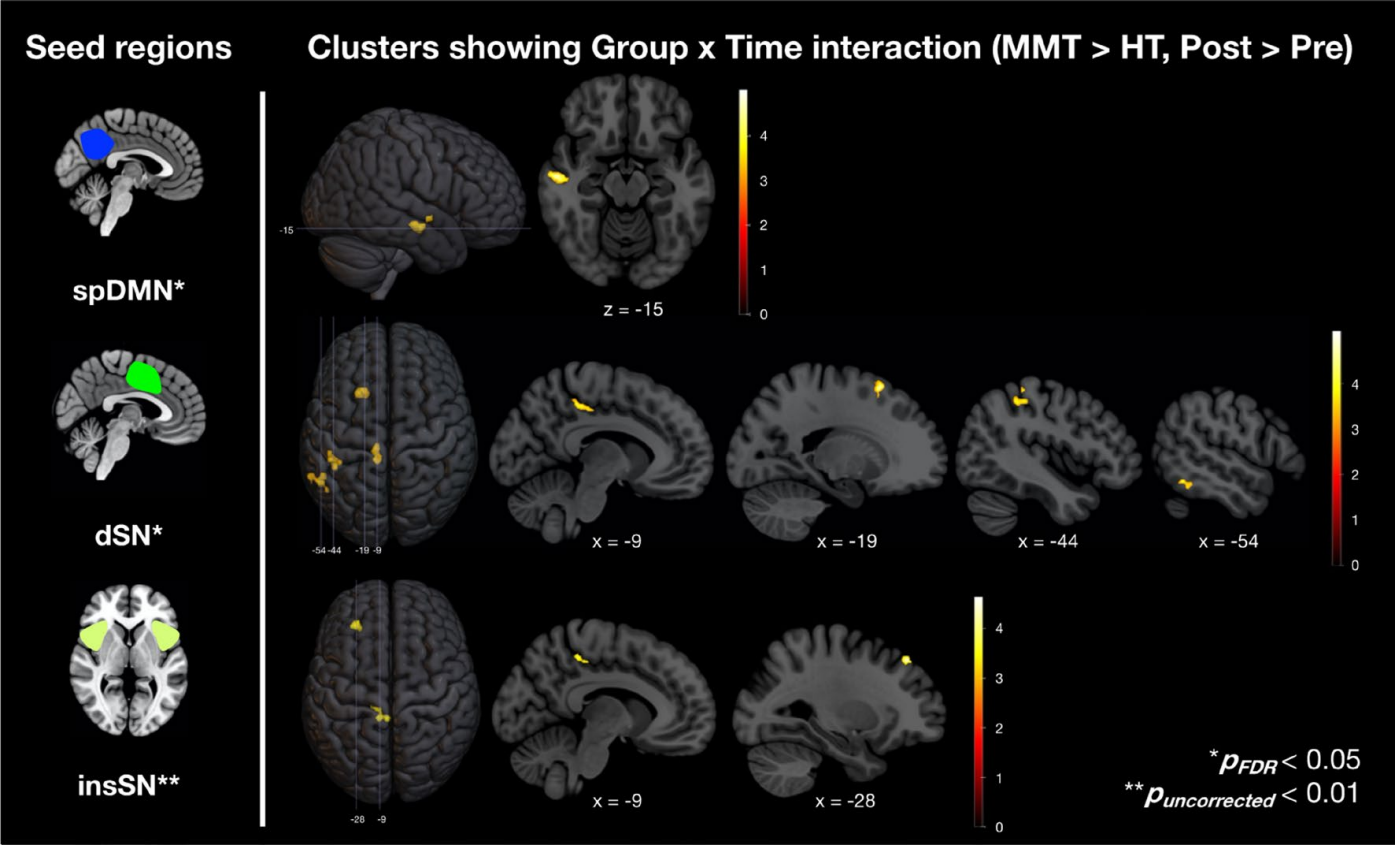
Seed region and the clusters showing group by time interactions as displayed on a Montreal Neurological Institute template taken from ICBM 152 Nonlinear atlases version 2009 (© 1993–2004 Louis Collins, McConnell Brain Imaging Centre, Montreal Neurological Institute, McGill University)^43^. Visualized with help of MRIcroGL v1.2.20210317 (https://www.nitrc.org/projects/mricrogl)^44^. *MMT* Mindfulness Meditation Training, *HT* Health Training, *spDMN* superior posterior default mode network, *dSN* dorsal salience network, *insSN* insular salience network).

Based on the results from analysis of dynamic functional connectivity showing MMT-associated changes in spDMN, dSN and insSN connectivity, the spatial maps of these network components were employed as seeds for a seed-based connectivity analysis. Using the spDMN seed, whole brain, FDR-corrected analysis revealed a cluster showing significant group by time interaction within the right middle temporal gyrus (MTG) (Fig. 4, Table 3). The dSN seed displayed an FDR-corrected group by time interaction with four clusters, within the left supramarginal gyrus (SMG), left premotor cortex (PMC), left fusiform gyrus (FFG) and dorsal posterior cingulate cortex (dPCC) (Fig. 4, Table 3). No clusters were found for the insSN seed at an FDR-corrected threshold.

**Table 3:** ANOVA results and peak activations of clusters with associated anatomic regions.

Seed region	Cluster region		*p*_FDR_	*p*_uncorrected_	*k*_*E*_	_*Tmax*_	Peak MNI-coordinates		
							*x*	*y*	*z*
spDMN	R	Middle temporal gyrus	0.002	< 0.001	104	5.18	56	− 12	− 16
dSN	L	Superior frontal gyrus	0.019	< 0.001	72	5.45	− 20	18	60
	L	Posterior cingulate cortex	0.045	0.001	53	4.43	− 10	− 36	46
	L	Inferior temporal gyrus	0.019	< 0.001	68	4.30	− 52	− 50	− 20
	L	Inferior parietal lobule	0.016	< 0.001	85	4.13	− 46	− 36	58
insSN	L	Middle frontal gyrus	0.243	0.006	36	4.90	− 28	38	48
	L	Posterior cingulate cortex	0.198	0.003	46	4.22	− 2	− 38	50

*k*_*E*_ Number of voxels in cluster, *T*_*max*_ Maximum value of T-statistic in cluster, *spDMN* superior posterior default mode network, *dSN* dorsal salience network, *insSN* insular salience network, *R* Right hemispheric, *L* Left hemispheric.

**Figure 5:**
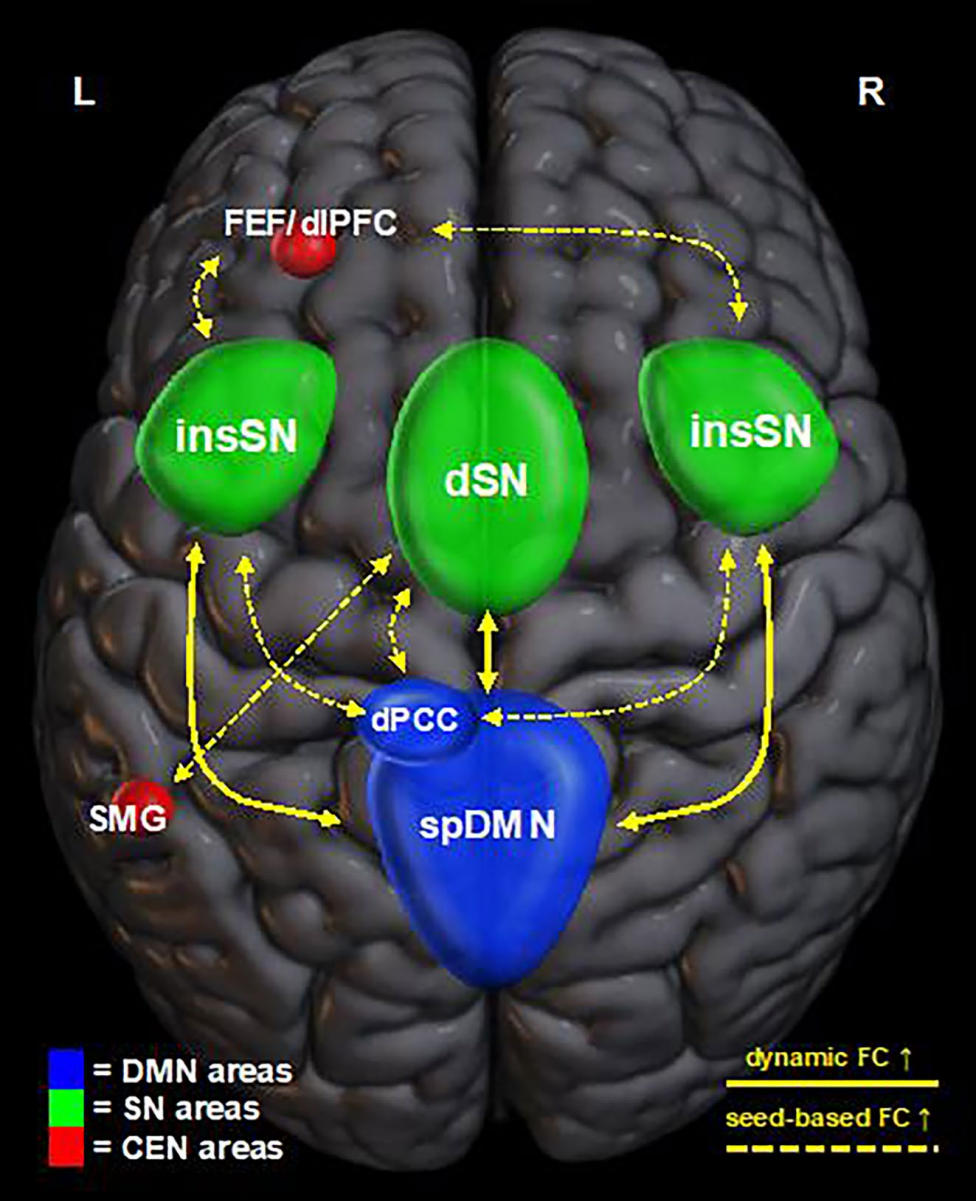
Schematic display of mindfulness-induced interaction patterns revealed through dynamic functional connectivity (FC) (solid lines) and seed-based FC analysis (dashed lines). Visualized with help of MRIcroGL v1.2.20210317 (https://www.nitrc.org/projects/mricrogl)^44^. Default mode network (DMN) associated areas are depicted in blue, salience network (SN) areas in green and central executive network (CEN) associated areas in red. Results from dynamic FC and seed-based FC reproduced highly similar interaction patterns between DMN and SN areas. Besides, SN regions showed increased FC with areas associated with the CEN. *FEF/dlPFC* Frontal eye field/dorsolateral prefrontal cortex, *insSN* insular salience network, *dSN* dorsal salience network, *dPCC* dorsal posterior cingulate cortex, *spDMN* superior posterior default mode network, *SMG* supramarginal gyrus).

An exploratory analysis using an uncorrected threshold of *p* < 0.01 exposed two clusters showing significant group by time interaction with the insSN seed. The first is situated at the border between the left frontal eye field (FEF) and left dorsolateral prefrontal cortex (dlPFC) and the second one was located within the dPCC (Fig. 4, Table 3). Remarkably, the second cluster was found to be almost in full alignment with the dPCC cluster discovered using the dSN seed. Moreover, belonging to the dPCC, these clusters share a functional–anatomic correlate with the spDMN. Thus, across both analyses, analogous interactions between two key regions of the SN (insSN/insula, dSN/ACC) and the DMN (spDMN/PCC) were observed (Fig. 5).

As prior research indicates a relevant interaction of age with meditation-related changes in the brain^45,46^, the second level analysis was repeated controlling for age. The results presented above did not change in consequence of this procedure.

## Discussion

In summary, these results demonstrate that one month of mindfulness meditation practice leads to the functional reorganization of large-scale brain networks in meditation-naïve subjects. Across different analyses, we found coherent responses of DMN–SN connectivity to MMT (Fig. 5). First, a sliding time window analysis revealed two subnetworks of the SN (dSN, insSN) mutually displaying connectivity increases with another subnetwork of the DMN (spDMN). Upon subsequently investigating whole brain connectivity using these network components as seeds, both subnetworks of the SN, once again, exhibited increased connectivity to a mutual area, the PCC. This region, in turn, is anatomically associated with the subnetwork of the DMN from our previous analysis. Although cluster and component did not spatially intersect, we consider the analogy across both analyses to be strong evidence for a mindfulness-mediated increase of interaction between the DMN and the SN.

This conclusion, however, shall first be validated against some objections. After all, analysis of static FC, which assesses changes in averaged connectivity over the whole duration of the scan, was not able to detect any alterations in inter-network connectivity. However, through different investigations, it was confirmed that connectivity in the brain during resting state is subject to dynamic fluctuations with more subtle connectivity phenomena occurring beyond the scope of static connectivity^47,48^. Discrepant results between the analyses of static and of dynamic FC might therefore be plausible, simultaneously reflecting an advantage specific to sliding time window analysis in identifying refined connectivity phenomena and, at the same time, potentially implying a limited intensity of the observed effects. It therefore poses the question whether we must consider these results strictly within the single state that statistical significance has been exclusive to or if general inferences are possible. That increases in FC between the DMN and SN do indeed exist throughout the whole measure is exemplified by Fig. 3 which depicts correlation values between these network pairs along the sliding time window. For both pairs, correlation values in participants who completed the MMT can be found to be substantially higher and almost continuously above those measured at baseline or in participants of the HT, allowing us to conclude the presence of increases independently of state. Further proof is provided by analyses of seed-based connectivity which also make use of averaged time courses and have revealed a complementary interaction pattern. While it must be taken into account that in order for these dynamics to appear we had to gradually lower the statistical threshold for one of the seed regions, we believe that the near alignment of both clusters and their location within a subregion of the PCC (which has specifically been shown to yield responsivity to SN-associated areas^49^) gives strong reason to assume their plausibility. Ultimately, it is the consistency of interaction patterns across both analyses that provides compelling evidence for assuming connectivity increases between the DMN and SN through mindfulness meditation.

To our knowledge, this is the first report of mindfulness-meditation induced increments in connectivity between the DMN and the SN on a comprehensive network-scale. Previous research targeting key regions of these networks has already demonstrated an increased connectivity between the PCC and ACC, a core hub of the SN, in experienced meditators compared to meditation-naïve controls^34^. In clinical trials, mindfulness-based interventions have been found to strengthen this connection in individuals suffering from post-traumatic stress disorder^50^ or breast cancer survivors affected with chronic neuropathic pain^51^ or even between the PCC and both the ACC and insular cortex in patients with generalized anxiety disorder^52^. Whereas these findings already unveiled partial responses of the DMN-SN relationship to mindfulness meditation, our results enable us to embed this hypothesis in a network-based paradigm which provides a well-suited environment to translate the cognitive processes accompanying meditation to neural activity. This is illustrated by pioneering work coming from Hasenkamp and colleagues^53^ which proposes a four-stage cognitive cycle surrounding the loss and resumption of focused attention (FA) while undergoing attention to breath meditation (as it is also a central feature of MMT). While undergoing fMRI, meditators were instructed to signal the moment they notice a loss of focus by pushing a button. This state of awareness was assumed to be preceded by a state of mind-wandering and to be followed by a shift of attention and its subsequent maintenance. By temporally dissecting the functional signal surrounding this indication, the authors were able to match these states to cortical activations. Interestingly, the associated activation patterns corresponded largely to networks of the triple network model: A loss of focused attention was linked to activation of areas pertaining to the DMN, gaining awareness of this with divisions associated with the SN and the shift and reuptake of attention was linked to activations of the dlPFC and SMG, key regions of the CEN. Regular practice of mindfulness meditation could therefore stimulate communication between the involved networks. Upon this evidence, we hypothesize that increased connectivity between the DMN and the SN reflects mindfulness meditators becoming more effectively aware of DMN-related processes such as mind-wandering or self-referential thoughts.

In addition to this, the SN seed regions displayed connectivity increases with two clusters within left-hemispheric core regions of the CEN, i.e., the dlPFC and the SMG. We did not observe these dynamics on a network level, possibly because the ICA only returned a right-sided CEN and the observed changes were restricted to the left hemisphere. Nevertheless, these findings logically complement correspondence to the framework described above and are further in line with general assumptions about the triple network model. Thus, they conceive an interplay in which the SN notices preponderance of the DMN and, upon this, initiates reinstalment of the CEN. Our results indicate that mindfulness meditation effectively enhances this interaction, potentially allowing regular practitioners to regain attention more efficiently thereby facilitating cognitive efforts such as sustained attention.

Further connectivity increases were observed between the spDMN seed and a cluster in the right middle temporal gyrus (MTG). Regional activation of the MTG has been shown to be less in experienced meditators^34^ and negatively correlated with dispositional mindfulness^54^ but, to our knowledge, information on training-related changes in regional activation and FC of the MTG is missing. Also pertaining to the DMN^55,56^, this region has been associated with language and thought^57^. Hence, potential interpretations include a shift of awareness towards language and thought, since the meditation sessions during the MMT were accompanied by verbal instructions. Another possible explanation could be that participants of the MMT were able to focus more effectively on the verbal instructions given for the resting state sequence. However, these inferences must be treated with caution and more research is needed to explore this interaction.

Opposing findings have been made in elderly participants of MBSR, where graph-based analysis revealed decreased nodal strength of the PCC, indicating less connectivity with nodes within other LSNs, including the SN, in consequence of the training^58^. As mentioned above, age has been shown to play a relevant role in the formation of meditation-related changes in the brain^45,46^. Therefore, it can be argued that these findings cannot necessarily be transferred to a younger population. In another study conducted by Lim and colleagues, dynamic FC in top performers of a breath counting task, which was interpreted to be representative of high trait mindfulness, was compared to dynamic FC of the bottom tertile^59^. Here, top performers spent more time in a state characterized by greater within-network connectivity of the DMN and task-positive networks, i.e., the SN and CEN, and showed greater anti-correlations between the DMN and task-positive networks^59^. However, spontaneous performance in a breath counting task does not directly translate to trait mindfulness. Instead, it can be argued that counting the number of breathings within a time span is much more representative for FA meditation than for open monitoring (OM) practices. Both of which are styles, which have been found to go along with distinct connectivity patterns: While for FA, decreased FC between the DMN and task-positive networks has been reported^60^, for OM increased FC has been observed^61^. Further evidence comes from a meta-analysis demonstrating for both FA and OM increased regional activation of regions involved in voluntary regulation of thought and action, such as the ACC, whereas decreased activation of areas associated with conceptual processing, such as the PCC was only observed for OM. The latter is in contradiction to the aforementioned findings of Lim and colleagues, where higher levels of mindfulness were linked to higher within-network connectivity of the DMN. Hence, it could be reasoned, that the results presented here are more influenced by the OM practice. This could further be related to the instruction given to participants for the resting state sequence of not engaging in “any specific trains of thoughts as much as possible”. Thus, the question arises whether the increases in FC between the DMN and SN were induced by participants of the MMT engaging in OM practice during the scan, rather than by a change in spontaneous brain activity. While these results still demonstrate distinct effects of MMT on brain function, future research should implement scanning conditions that specifically allow for the distinction between meditation-related processes and spontaneous brain activity.

In brief, FA practice can be associated to increased activation in areas associated with cognitive control (which are also associated with the SN) and decreased inter-connectivity of the triple network model, while OM practice was associated with decreased activation of DMN regions and increases in inter-network connectivity. This could indicate that, through FA practice, neural activity is primarily concentrated in control regions and, therefore, requires less interaction between networks, whereas OM practice leads to less activation of the DMN and, the consequential shift of activation requires an increase of inter-network connectivity.

Among the first to report decreases in DMN activity during meditation practice were Brewer and colleagues^34^. Comparing brain activation, they found less activation of central nodes of the DMN, particularly the PCC, in experienced meditators versus meditation-naïve controls^34^. These findings have been supported using other modalities. Within the PCC, for example, amplitudes of low frequency fluctuations (ALFF), which are thought to reflect spontaneous brain activity, were observed to be lower in long-term meditators^62^ and to decline after MBSR^63^. Additionally, magnetencephalographic studies have unveiled distinct connecticity patterns of the PCC that relate to different styles of meditation, illustrating how crucial this region is to meditation^60^. Interestingly, grey matter density of the PCC has been found to increase following MBSR^64^. Generally, alterations in grey matter density are conceived to represent adaptive changes to performative demands^65^. This would somewhat oppose the findings discussed before which suggested regional deactivation of the PCC in relation to meditation practice. Hence, we assume that the interaction between brain function and meditation is of high complexity and involves multiple factors.

Various studies have outlined the impact of brief meditation interventions on structural and functional connectivity as well as on behavioral outcomes^66–68^. Others have identified differences in brain structure and functional connectivity unique to long-term meditators^69,70^. This gives grounds to conclude that, aside from the style of meditation, intervention-related changes in spontaneous brain activity and brain activity during the state of meditation as well as long-term practice of meditation each go along with individual neural signatures that have yet to be fully deciphered.

Finally, we would like to acknowledge, that changes in brain function were not accompanied by increases of self-reported mindfulness which could indicate that the intervention did not have an impact on subjective levels of mindfulness. However, this is not uncommon and it has been argued that roughly half of the studies incorporating mindfulness-based interventions do not observe changes in subjective mindfulness^4^. Moreover, as results from the Bayesian ANOVA indicated inconclusive evidence, we reason that the effect size of the present study was probably not high enough to detect changes in subjective levels of mindfulness with the present sample size and more data, i.e., larger sample sizes, might be needed to successfully capture effects of our meditation training on subjective levels of mindfulness. Another possible explanation includes the training intensity, as dosage-specific effects of MMT on self-reported mindfulness can be assumed.

Hence, future research should incorporate larger samples and account for different stages of training intensity, i.e., through prospective cohort designs with repeated measures along the course of the training. It would further be interesting to investigate the perseverance of effects beyond a regular meditation practice, as these are presumably going to subside. And with respect to the model we have proposed, future work should explicitly target the nexus of network-based neural activation and its concomitant cognitive processes in order to eventually attain a translational perspective on the abundance of beneficial effects attributed to mindfulness meditation.

## Conclusion

Taken together, these findings indicate that, among multiple LSNs, one month of MMT leads to increased connectivity between networks of the triple network model (DMN, SN, CEN). This interaction corresponds well to cyclical cortical activations that previous research has linked to cognitive processes during a state of meditation. Based on this framework, we hypothesize that MMT allows the SN to modulate between the DMN and CEN more effectively in favor of the CEN. On a cognitive level, this could allow regular practitioners to notice a loss of attention more effectively and might facilitate its subsequent re-instalment. However, further studies are needed to support this hypothesis with behavioral data and to estimate the influence of training intensity on this evidence.

## Methods

### Participants

Participants were recruited through advertisements on hospital bulletin boards, online, and through word-of-mouth. Potential candidates were screened for the presence of psychiatric or neurological diseases using the Mini-international neuropsychiatric interview (M.I.N.I.)^71^ and, if negative, were subsequently deemed eligible for participation when meeting the following criteria: (1) Age between 18 and 60, (2) general MRI suitability, (3) right-handedness, (4) meditation experience of less than three meditation sessions within the past year or less than ten meditation sessions within the entire life span, and (5) abstinence from psychotropic drugs. All participants provided written informed consent and received a monetary compensation for their participation. The study was approved by the Ethics Committee of Klinikum Rechts der Isar, Technische Universität München. All methods were carried out in accordance with relevant guidelines and regulations.

### Procedure

This study was designed as a pseudo-randomized, controlled and parallel trial to investigate the effects of mindfulness meditation on brain function. All subjects enrolled were single-blindedly (subject only) allocated to equally sized groups of either 31 days of MMT or 31 days of a HT and underwent magnetic resonance imaging (MRI) and questionnaire-based assessment of subjective levels of mindfulness before and after the intervention. Simple randomization was performed after the first visit. However, due to the relatively small sample size, the age and gender equilibrium eventually had to be actively maintained by the investigators. Mindfulness levels and resting state functional connectivity were specified as primary outcomes. Prior to the experiment, no information about study’s main aims, aside from promoting health awareness, was revealed to participants. Both training programs were accessible via an online platform and consisted of 31 sessions, delivered in daily portions of 10–15-min courses. Starting and finishing with a video clip, every third session contained a video, others were presented as audio recordings.

The MMT was developed in close cooperation with Britta Hölzel (B.H.) who is a certified MBSR instructor and provided an introduction to the theoretical framework of mindfulness as well as daily guided meditation exercises. The HT was designed to be an informative and active control intervention and contained excerpts from popular science broadcasting formats, covering a broad range of health-related topics. Care was taken, that topics were unrelated to mindfulness or meditation in general. For a detailed description of training contents see Supplement, Table S2. Participants needed to complete at least 23 training sessions to be included for the final analysis.

**Figure 6:**
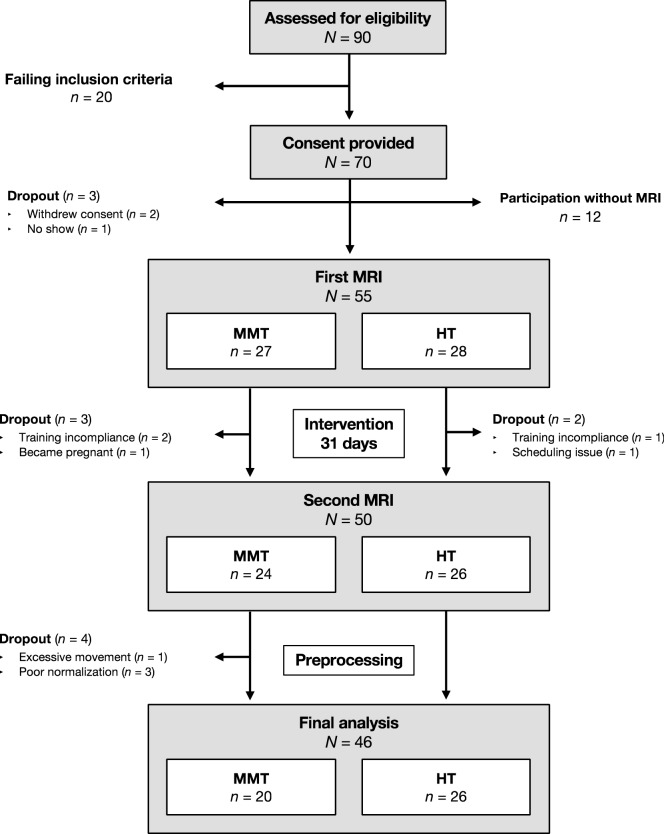
Data acquisition scheme and participant flow chart. *MMT* Mindfulness Meditation Training, *HT* Health Training.

A sample size of 30 subjects per group was determined as it allows for assessing strong effects (d = 0.8) with a power of approximately 0.86 via *t*-tests or Pearson correlation (independent samples, two-sided *t*-tests, β/α ratio = 1). A similar power is achieved for strong group effects with an analysis of variance with repeated measures (γ = 0.94–0.96). 55 subjects (27 female) were eventually recruited and completed the first scanning appointment, 50 (25 female) were available for the second appointment and, after preprocessing, data from 46 (23 female) participants were included for the final analysis. An acquisition scheme and participant flow chart is illustrated in **Fig. ****6**. The data acquisition process took place between August 2017 and September 2019.

To verify the success of the pseudo-randomization process, final samples were compared for demographic characteristics, measuring intervals, and average number of sessions completed using t-tests for independent samples or c^2^-tests, respectively. In order to account for possible impacts of pre-existing health-related behavior on training-specific effects, lifestyle characteristics, including Body Mass Index (BMI), cigarette and alcohol consumption as well as physical exercise, were also assessed and groups compared for potential differences.

### Assessment of self-reported mindfulness

All participants were asked to complete the Mindful Attention and Awareness Scale (MAAS)^72^ during both of their scanning appointments. The MAAS is a 15-item, self-report scale measuring the intensity of mindfulness in daily life (e.g., “I find it difficult to stay focused on what’s happening in the present”) and has been shown to be generally susceptible to training effects^40,73^. High scores indicate high levels of subjective mindfulness. Data from one participant was excluded due to incomplete information. One outlier (> *M* + 3 *SD*) was identified and the associated participant was discarded for the behavioural analysis.

To investigate the effects of MMT vs. HT, MAAS scores were entered into a 2 × 2 mixed effects ANOVA using SPSS v26.0, treating group as between-subject factor and time as within-subject factor. Results were thresholded at *p* < 0.05.

In addition to this, a Bayesian ANOVA was conducted to investigate training effects. In frequentist statistics, evidence is gathered by accepting an alternative hypothesis (*H*_*A*_, assuming the presence of effects) over the rejection of a null hypothesis (*H*_*0*_, assuming the absence of effects), limiting inferences to evidence deriving from *H*_*A*_. In Bayesian statistics, these hypotheses represent different models whose individual likelihood ratios can be quantified thus allowing for the distinction between evidence in favor of or against either hypothesis and, moreover, for identifiying inconclusive evidence. For this purpose, the relative probability of each model before and after observing the data is assessed and the ratio of their odds is subsumed into a Bayes Factor (*BF*). The *BF*_*10*_ is a continuous measure with a range from 0 to + ∞ and indicates the relative likelihood of *H*_*A*_ compared to the likelihood of *H*_*0*_, whereas the *BF*_*01*_ indicates the inverse and corresponds to the reciprocal numerical value. A *BF*_*10*_ > 3 provides moderate evidence in favor of *H*_*A*_ and a *BF*_*10*_ > 10 provides strong evidence in favor of *H*_*A*_. If the *BF*_*10*_ is between 1/3 and 3, the evidence is considered inconclusive and further data is required. The same classification is applied to the *BF*_*01*_ with evidence pertaining to *H*_*0*_, e.g., a *BF*_*01*_ > 3 corresponds to a *BF*_*10*_ < 1/3 and indicates that the data are at least three times as likely to occur under *H*_*0*_ than under *H*_*A*_. Bayesian statistics were computed using JASP (https://jasp-stats.org) and default Cauchy priors (fixed effects *r* = 0.5, random effects *r* = 1, and covariates *r* = 0.354)^74,75^.

### MRI data acquisition

All imaging data were collected on a 3 T Philips MRI scanner with a 32-channel head coil at Klinikum Rechts der Isar, München, Germany.

T_2_*-weighted resting state functional images were acquired using echo planar imaging (EPI) with the following scanning parameters: Multiband (MB) factor 2, repetition time (TR) 2.7 s, echo time (TE) 33 ms and flip angle 90°. The field of view (FOV) was set to (192 × 192 × 141) mm^3^, corresponding to a matrix size of 96 × 96 with 64 axial slices of 2 × 2 × 2 mm^3^ large isotropic voxels. 200 volumes were obtained over the course of 9 min. Subjects were instructed to keep eyes closed and to refrain from engaging in any trains of thought as much as possible.

For normalization and structural reference, high-resolution T_1_-weighted anatomical images were acquired using a magnetization-prepared rapid acquisition gradient echo (MPRAGE) sequence with the following scanning parameters: TR 11 ms, TE 5.2 ms and flip angle 8°. 230 axial AC-PC slices encompassing a 384 × 384 matrix of 0.7 × 0.7 × 0.7 mm^3^ large isotropic voxels were obtained. All anatomical images underwent clinical inspection by a medical specialist prior to further analysis.

### Preprocessing

Preprocessing of imaging data was conducted using SPM 12 (The Wellcome Centre for Human Neuroimaging; http://www.fil.ion.ucl.ac.uk/spm). The preprocessing pipeline was created in correspondence to the work of Allen and colleagues who performed group-ICA on resting state fMRI data of 603 healthy participants and, thereby, provided a baseline for multivariate comparison of resting-state networks^76^. First, the anatomical image was coregistered to the mean functional image and segmented into tissue probability maps, which were then used to create a group specific DARTEL template^77^. Using these templates, the realigned functional timeseries was normalized to MNI space and smoothed using a 4 × 4 × 4 mm^3^ full width at half maximum Gaussian Kernel. The first five volumes were discarded to allow for stabilization of the magnetic field. The scanning protocol defined a TR of 2.7 s and had a MB factor of 2. This makes it possible to simultaneously acquire multiple slices within a short time period which reduces the temporal difference between individual slices. Hence, to limit data manipulation while following evidence suggesting its negligible influence on quality of resting state connectivity analyses^78^, no slice time correction was performed. Customised scripts from DPABI v5.1 were used to assess framewise displacement (FD Jenkinson) and one subject was removed due to a mean value greater than 0.3^79,80^.

### Independent component analysis

To detect ICNs, the preprocessed data were entered to an ICA using GIFT toolbox v4.0b (http://icatb.sourceforge.net). A high-model-order approach with 75 components was pursued since this number was shown to successfully identify fine-grained components corresponding to known functional–anatomical network divisions^76,81^. Data from all participants were concatenated and reduced through a two-step principal component analysis. Then, the Infomax algorithm implemented in MATLAB was applied to estimate a total of 75 independent components (ICs). To ensure component stability, this was repeated 20 times using the Icasso software package^82^. Finally, the averaged components were back reconstructed into single subject space using the group ICA algorithm, resulting in spatial maps representing voxel-specific z-scores of within-component FC and component-specific time courses of blood oxygenation level dependent (BOLD) signal fluctuations. Possible networks of interest were then identified by performing multiple spatial regression of these maps with an established set of templates^76^ and additional visual classification. To be considered eligible for further analysis, networks had to provide a stability index (iq) of at least 0.8. This index ranges from 0 to 1 and reflects the stability of a component throughout multiple (in this case 20) repetitions of the Infomax ICA algorithm^82^. The final selection was based upon our initial hypothesis (as mentioned above: networks corresponding to the triple network model and to areas associated with attention control, emotion regulation, and self-awareness as well as sensorimotor regions) and included a total of 12 network components, subdivided into 6 large-scale networks (Fig. 1).

#### Static functional connectivity

To investigate effects of MMT vs. HT on FC within networks, the spatial maps of the 12 selected network components of interest were entered into a 2 × 2 mixed effects ANOVA in SPM, treating group as between-subject factor and time as within-subject factor. Network-specific grey matter masks were applied and results were thresholded at *p* < 0.05, false-discovery-rate (FDR) corrected for multiple comparisons.

To investigate effects of MMT vs. HT on FC between networks, MANCOVAN toolbox v1.0b as integrated into GIFT was used. First, each ICs time course was filtered (high cutoff 0.15 Hz), de-trended and de-spiked. For each subject and measurement time point, Pearson correlation coefficients were calculated between time courses of each of the 12 selected network components of interest. The resulting 78 values were Fisher r-to-z transformed to assure normal distribution for second level analysis. Z-scores were then entered into a 2 × 2 mixed effects ANOVA in SPSS v26.0, treating group as between-subject factor and time as within-subject factor. Results were thresholded at *p* < (0.05/78 =) 0.0006, Bonferroni-corrected for multiple comparisons.

#### Dynamic functional connectivity

To account for dynamics of functional connectivity^47,48,83^ and the contingency of further temporal refinement of inter-network connectivity, sliding time window analyses were conducted using the temporal dFNC toolbox integrated into GIFT which follows the approach established by Allen and colleagues^84^. A sliding time window with a width of 30 TRs was applied to the filtered, de-trended and de-spiked time course of each of the 12 network components of interest and shifted in steps of 1 TR, providing a total of 165 windows. A Gaussian σ of 3 TRs was used to taper the edges of the otherwise rectangular window. For each window, Pearson correlation coefficients were computed between network pairs. Then, time windows are sub-sampled for each subject and windows with local maxima in variance of functional connectivity are identified. These subject exemplars are then used to automatically estimate a number of *k*-means clusters (*k* = 5) using gap statistic and silhouette algorithms, as integrated into the dFNC toolbox. Finally, the *k*-means algorithm is applied to windowed covariance matrices to identify reoccurring FC patterns (centroids) across all subjects and time points. These patterns are later considered states of connectivity that occur reproducibly across subjects.

To investigate the effects of MMT vs. HT, connectivity states (state-specific Fisher r-to-z transformed correlation between time courses of networks components), mean dwell time (average time spent within a specific state), and total number of states occurring (cumulative number of individual states entered along time course) were compared within groups using paired t-tests. Baseline measures were compared using t-tests for independent samples to exclude pre-existing differences and thus allow for better comparison of the groups. With respect to the number of multiple comparisons and connectivity states, an FDR- and Bonferroni-corrected threshold of *p*_FDR_ < (0.05/5 =) 0.01 was applied to the results.

### Seed-based connectivity

As results from dynamic functional connectivity analysis indicated MMT associated changes in DMN and SN connectivity, subsequent seed-based connectivity analyses were conducted treating the spatial maps of ICNs subject to these alterations as seeds in order to asses whole brain connectivity of these networks. Using CONN Toolbox v20.b^85^, additional preprocessing steps performed on preprocessed data included denoising by regressing out white matter and cerebrospinal fluid using CompCor^86^ and then filtering time courses with a bandpass filter of 0.01 to 0.1 Hz, as well as de-trending and de-spiking. To create first level connectivity maps, Pearson correlation coefficients were computed between the average time courses of voxels within each ROI and every brain voxel and transformed to z-scores. For the second level analysis, these maps were entered into a 2 × 2 mixed ANOVA, treating group as between-subject factor and time as within-subject factor. Results were simultaneously contrasted at MMT > HT and Post > Pre and thresholded at *p* < 0.05, FDR-corrected for multiple comparisons.

## Supplementary Information

Supplementary Tables.

## Data Availability

Data have been made publicly available via the Open Science Framework at https://doi.org/10.17605/osf.io/rz3hs.

## References

[1] Kabat-Zinn J (1990). Full Catastrophe Living: Using the Wisdom of Your Body and Mind to Face Stress, Pain, and Illness.

[2] Bishop SR (2004). Mindfulness: A proposed operational definition. Clin. Psychol. Sci. Pr..

[3] Chiesa A, Calati R, Serretti A (2011). Does mindfulness training improve cognitive abilities? A systematic review of neuropsychological findings. Clin. Psychol. Rev..

[4] Creswell JD (2017). Mindfulness interventions. Annu. Rev. Psychol..

[5] Mrazek MD, Franklin MS, Phillips DT, Baird B, Schooler JW (2013). Mindfulness training improves working memory capacity and GRE performance while reducing mind wandering. Psychol. Sci..

[6] Zeidan F, Johnson SK, Diamond BJ, David Z, Goolkasian P (2010). Mindfulness meditation improves cognition: Evidence of brief mental training. Conscious Cogn..

[7] Khoury B, Sharma M, Rush SE, Fournier C (2015). Mindfulness-based stress reduction for healthy individuals: A meta-analysis. J. Psychosom. Res..

[8] Sharma M, Rush SE (2014). Mindfulness-based stress reduction as a stress management intervention for healthy individuals. Evid. Based Complement. Alternat. Med..

[9] Goldberg SB (2018). Mindfulness-based interventions for psychiatric disorders: A systematic review and meta-analysis. Clin. Psychol. Rev..

[10] Zeidan F (2015). Mindfulness meditation-based pain relief employs different neural mechanisms than placebo and sham mindfulness meditation-induced analgesia. J. Neurosci..

[11] Zeidan F, Vago DR (2016). Mindfulness meditation-based pain relief: a mechanistic account. Ann. N. Y. Acad. Sci..

[12] Goyal M (2014). Meditation programs for psychological stress and well-being. JAMA Intern. Med..

[13] Rodrigues MF, Nardi AE, Levitan M (2017). Mindfulness in mood and anxiety disorders: A review of the literature. Trends Psychiatr. Psychother..

[14] Boyd JE, Lanius RA, McKinnon MC (2018). Mindfulness-based treatments for posttraumatic stress disorder: A review of the treatment literature and neurobiological evidence. J. Psychiatry Neurosci..

[15] Moreno C (2020). How mental health care should change as a consequence of the COVID-19 pandemic. Lancet Psychiatry.

[16] Richter D, Riedel-Heller S, Zürcher SJ (2021). Mental health problems in the general population during and after the first lockdown phase due to the SARS-Cov-2 pandemic: Rapid review of multi-wave studies. Epidemiol. Psychiatr. Sci..

[17] Tang Y-Y, Hölzel BK, Posner MI (2015). The neuroscience of mindfulness meditation. Nat. Rev. Neurosci..

[18] Bahmani Z (2019). Prefrontal contributions to attention and working memory. Curr. Top. Behav. Neurosci..

[19] van Veen VC, Cameron S (2002). The anterior cingulate as a conflict monitor: fMRI and ERP studies. Physiol. Behav..

[20] Allen M (2012). Cognitive-affective neural plasticity following active-controlled mindfulness intervention. J. Neurosci..

[21] Hölzel BK (2007). Differential engagement of anterior cingulate and adjacent medial frontal cortex in adept meditators and non-meditators. Neurosci. Lett..

[22] Doll A (2016). Mindful attention to breath regulates emotions via increased amygdala-prefrontal cortex connectivity. Neuroimage.

[23] Murakami H (2015). Neural networks for mindfulness and emotion suppression. PLoS ONE.

[24] Taren AA (2015). Mindfulness meditation training alters stress-related amygdala resting state functional connectivity: A randomized controlled trial. Soc. Cogn. Affect. Neurosci..

[25] Desbordes G (2012). Effects of mindful-attention and compassion meditation training on amygdala response to emotional stimuli in an ordinary, non-meditative state. Front. Hum. Neurosci..

[26] Kral TRA (2018). Impact of short- and long-term mindfulness meditation training on amygdala reactivity to emotional stimuli. Neuroimage.

[27] Lutz J (2014). Mindfulness and emotion regulation: An fMRI study. Soc. Cogn. Affect Neurosci..

[28] Buckner RL, Andrews-Hanna JR, Schacter DL (2008). The brain's default network. Ann. N. Y. Acad. Sci..

[29] Gusnard DA, Akbudak E, Shulman GL, Raichle ME (2001). Medial prefrontal cortex and self-referential mental activity: Relation to a default mode of brain function. PNAS.

[30] Philippi CL (2021). Lesion network mapping demonstrates that mind-wandering is associated with the default mode network. J. Neurosci. Res..

[31] Raichle ME (2001). A default mode of brain function. PNAS.

[32] Mohan A (2016). The significance of the defualt mode network (DMN) in neurological and neuropsychiatric disorders: A review. YJBM.

[33] Zhou HX (2020). Rumination and the default mode network: Meta-analysis of brain imaging studies and implications for depression. Neuroimage.

[34] Brewer JA (2011). Meditation experience is associated with differences in default mode network activity and connectivity. PNAS.

[35] Menon V (2011). Large-scale brain networks and psychopathology: a unifying triple network model. Trends Cogn. Sci..

[36] Sridharan D, Levitin DJ, Menon V (2008). A critical role for the right fronto-insular cortex in switching between central-executive and default-mode networks. PNAS.

[37] Bilevicius E, Smith SD, Kornelsen J (2018). Resting-state network functional connectivity patterns associated with the mindful attention awareness scale. Brain Connect..

[38] Parkinson TD, Kornelsen J, Smith SD (2019). Trait mindfulness and functional connectivity in cognitive and attentional resting state networks. Front. Hum. Neurosci..

[39] Doll A, Hölzel BK, Boucard CC, Wohlschläger AM, Sorg C (2015). Mindfulness is associated with intrinsic functional connectivity between default mode and salience networks. Front. Hum. Neurosci..

[40] Kilpatrick LA (2011). Impact of mindfulness-based stress reduction training on intrinsic brain connectivity. Neuroimage.

[41] Fahmy R (2019). Mindfulness-based therapy modulates default-mode network connectivity in patients with opioid dependence. Eur. Neuropsychopharm..

[42] Bauer CCC (2020). Mindfulness training preserves sustained attention and resting state anticorrelation between default-mode network and dorsolateral prefrontal cortex: A randomized controlled trial. Hum. Brain Mapp..

[43] Fonov V (2011). Unbiased average age-appropriate atlases for pediatric studies. Neuroimage.

[44] Rorden C, Brett M (2000). Stereotaxic display of brain lesions. Behav. Neurol..

[45] Guidotti R, Del Gratta C, Perrucci MG, Romani GL, Raffone A (2021). Neuroplasticity within and between functional brain networks in mental training based on long-term meditation. Brain Sci..

[46] Luders E, Cherbuin N, Gaser C (2016). Estimating brain age using high-resolution pattern recognition: Younger brains in long-term meditation practitioners. Neuroimage.

[47] Chang C, Glover GH (2010). Time–frequency dynamics of resting-state brain connectivity measured with fMRI. Neuroimage.

[48] Hutchison RM, Womelsdorf T, Gati JS, Everling S, Menon RS (2013). Resting-state networks show dynamic functional connectivity in awake humans and anesthetized macaques. Hum. Brain Mapp..

[49] Leech R, Braga R, Sharp DJ (2012). Echoes of the brain within the posterior cingulate cortex. J. Neurosci..

[50] King AP (2016). Altered default mode network (DMN) resting state functional connectivity following a mindfulness-based exposure therapy for posttraumatic stress disorder (PTSD) in combat veterans of Afghanistan and Iraq. Depress Anxiety.

[51] Smith AM (2021). Mindfulness-based stress reduction alters brain activity for breast cancer survivors with chronic neuropathic pain: Preliminary evidence from resting-state fMRI. J Cancer Surviv.

[52] Zhao XR (2019). Mindfulness-based cognitive therapy is associated with distinct resting-state neural patterns in patients with generalized anxiety disorder. Asia-Pac. Psychiatry.

[53] Hasenkamp W, Wilson-Mendenhall CD, Duncan E, Barsalou LW (2012). Mind wandering and attention during focused meditation: A fine-grained temporal analysis of fluctuating cognitive states. Neuroimage.

[54] Way BM, Creswell JD, Eisenberger NI, Lieberman MD (2010). Dispositional mindfulness and depressive symptomatology: Correlations with limbic and self-referential neural activity during rest. Emotion.

[55] Margulies DS (2016). Situating the default-mode network along a principal gradient of macroscale cortical organization. Proc. Natl. Acad. Sci. USA.

[56] Yeo BT (2011). The organization of the human cerebral cortex estimated by intrinsic functional connectivity. J. Neurophysiol..

[57] Smallwood J (2021). The default mode network in cognition: A topographical perspective. Nat. Rev. Neurosci..

[58] Cotier FA, Zhang R, Lee TMC (2017). A longitudinal study of the effect of short-term meditation training on functional network organization of the aging brain. Sci. Rep..

[59] Lim J, Teng J, Patanaik A, Tandi J, Massar SAA (2018). Dynamic functional connectivity markers of objective trait mindfulness. Neuroimage.

[60] Marzetti L (2014). Magnetoencephalographic alpha band connectivity reveals differential default mode network interactions during focused attention and open monitoring meditation. Front. Hum. Neurosci..

[61] Raffone A (2019). Toward a brain theory of meditation. Prog. Brain Res..

[62] Berkovich-Ohana A, Harel M, Hahamy A, Arieli A, Malach R (2016). Alterations in task-induced activity and resting-state fluctuations in visual and DMN areas revealed in long-term meditators. Neuroimage.

[63] Yang CC (2019). Alterations in brain structure and amplitude of low-frequency after 8 weeks of mindfulness meditation training in meditation-naive subjects. Sci. Rep..

[64] Hölzel BK (2011). Mindfulness practice leads to increases in regional brain gray matter density. Psychiatry Res. Neuroimaging.

[65] Ilg R (2008). Gray matter increase induced by practice correlates with task-specific activation: A combined functional and morphometric magnetic resonance imaging study. J. Neurosci..

[66] Tang Y-Y, Lu Q, Fan M, Yang Y, Posner MI (2012). Mechanisms of white matter changes induced by meditation. PNAS.

[67] Tang Y-Y, Tang Y, Tang R, Lewis-Peacock JA (2017). Brief mental training reorganizes large-scale brain networks. Front. Syst. Neurosci..

[68] Zhang H, Zhang A, Liu C, Xiao J, Wang K (2021). A brief online mindfulness-based group intervention for psychological distress among chinese residents during COVID-19: A pilot randomized controlled trial. Mindfulness.

[69] Gard T (2015). Greater widespread functional connectivity of the caudate in older adults who practice kripalu yoga and vipassana meditation than in controls. Front. Hum. Neurosci..

[70] Sato JR (2012). Brain imaging analysis can identify participants under regular mental training. PLoS ONE.

[71] Sheehan DVL (1998). The mini-international neuropsychiatric interview (M.I.N.I.): The development and validation of a structured diagnostic psychiatric interview for DSM-IV and ICD-10. J. Clin. Psychiatr..

[72] Brown KW, Ryan RM (2003). The benefits of being present: Mindfulness and its role in psychological well-being. J. Pers. Soc. Psychol..

[73] Chambers R, Lo BCY, Allen NB (2008). The impact of intensive mindfulness training on attentional control, cognitive style, and affect. Cogn. Ther. Res..

[74] Keysers C, Gazzola V, Wagenmakers E-J (2020). Using Bayes factor hypothesis testing in neuroscience to establish evidence of absence. Nat. Neurosci..

[75] van den Bergh D (2019). A tutorial on conducting and interpreting a Bayesian ANOVA in JASP. PsyArXiv.

[76] Allen EA (2011). A Baseline for the multivariate comparison of resting-state networks. Front. Syst. Neurosci..

[77] Ashburner J (2007). A fast diffeomorphic image registration algorithm. Neuroimage.

[78] Wu CW (2011). Empirical evaluations of slice-timing, smoothing, and normalization effects in seed-based, resting-state functional magnetic resonance imaging analyses. Brain Connect..

[79] Power JD, Barnes KA, Snyder AZ, Schlaggar BL, Petersen SE (2012). Spurious but systematic correlations in functional connectivity MRI networks arise from subject motion. Neuroimage.

[80] Yan C-G, Wang X-D, Zuo X-N, Zang Y-F (2016). DPABI: Data processing & analysis for (resting-state) brain imaging. Neuroinformatics.

[81] Abou Elseoud A (2011). Group-ICA model order highlights patterns of functional brain connectivity. Front. Syst. Neurosci..

[82] Himberg J, Hyvärinen A, Esposito F (2004). Validating the independent components of neuroimaging time series via clustering and visualization. Neuroimage.

[83] Kiviniemi V (2011). A sliding time-window ICA reveals spatial variability of the default mode network in time. Brain Connect..

[84] Allen EA (2014). Tracking whole-brain connectivity dynamics in the resting state. Cereb. Cortex.

[85] Whitfield-Gabrieli S, Nieto-Castanon A (2012). Conn: A functional connectivity toolbox for correlated and anticorrelated brain networks. Brain Connect..

[86] Behzadi Y, Restom K, Liau J, Liu TT (2007). A component based noise correction method (CompCor) for BOLD and perfusion based fMRI. Neuroimage.

