# Supplementary material for: Mindfulness meditation increases default mode, salience, and central executive network connectivity
Source: Sci Rep. 2022 Aug 2;12:13219. doi: 10.1038/s41598-022-17325-6 (PMC9346127; doi:10.1038/s41598-022-17325-6)
Supplement: Supplementary file 1 — Supplementary Tables. [file 41598_2022_17325_MOESM1_ESM.doc]

**Supplementary material**

|  |  | |  |  | Peak MNI-coordinates | | |
| --- | --- | --- | --- | --- | --- | --- | --- |
| Network | Region | | *k*_E_ | *t*_max_ | x | y | z |
| aDMN | Bi | anterior cingulate cortex | 6330 | 29.64 | 0 | 38 | 16 |
|  | Bi | middle cingulate cortex | 175 | 9.36 | 0 | -14 | 38 |
| dpDMN | Bi | posterior cingulate cortex | 977 | 12.72 | -2 | -22 | 28 |
|  | Bi | precuneus | 3290 | 24.47 | 14 | -64 | 28 |
|  | L | inferior parietal lobule | 225 | 9.66 | -34 | -58 | 42 |
| spDMN | Bi | precuneus | 5642 | 34.15 | 0 | -56 | 30 |
|  | L | angular gyrus | 1336 | 15.55 | -40 | -68 | 32 |
|  | Bi | superior frontal gyrus | 461 | 13.02 | 0 | 56 | 20 |
|  | Bi | cerebellum | 121 | 11.48 | 4 | -56 | -50 |
|  | R | inferior frontal gyrus | 83 | 8.00 | 42 | 18 | 28 |
| ipDMN | Bi | posterior cingulate cortex | 6201 | 31.84 | 4 | -56 | 12 |
|  | R | middle frontal gyrus | 79 | 7.72 | 24 | 32 | 38 |
| vSN | Bi | anterior cingulate cortex | 3559 | 25.63 | 6 | 28 | 32 |
|  | L | insular cortex | 293 | 8.04 | -50 | 14 | -8 |
|  | R | insular cortex | 289 | 11.70 | 50 | 14 | -10 |
|  | L | middle frontal gyrus | 361 | 10.41 | -22 | 38 | 34 |
|  | L | inferior frontal gyrus | 199 | 9.26 | -44 | 16 | 30 |
|  | R | inferior frontal gyrus | 131 | 8.78 | 42 | 8 | 28 |
| dSN | Bi | supplementary motor area | 8455 | 33.89 | -2 | 8 | 42 |
|  | L | insular cortex | 646 | 24.89 | -56 | 8 | -2 |
|  | R | insular cortex | 607 | 13.44 | 50 | 6 | -2 |
|  | L | precentral gyrus | 95 | 11.37 | -20 | -28 | 58 |
|  | R | supramarginal gyrus | 235 | 11.19 | 50 | -30 | 24 |
|  | L | supramarginal gyrus | 236 | 10.73 | -48 | -30 | 20 |
|  | R | precentral gyrus | 67 | 7.24 | 22 | -26 | 60 |
|  | R | cerebellum | 61 | 8.01 | 28 | -58 | -24 |
|  | L | middle frontal gyrus | 92 | 7.77 | -32 | 44 | 34 |
| insSN | R | insular cortex | 2878 | 27.50 | 38 | 22 | -2 |
|  | L | insular cortex | 2296 | 25.70 | -42 | 12 | -2 |
|  | Bi | supplementary motor area | 1352 | 13.90 | -2 | 26 | 44 |
|  | Bi | anterior cingulate cortex | 430 | 10.83 | 4 | 46 | 0 |
|  | R | inferior frontal gyrus | 104 | 7.58 | 40 | 8 | 34 |
| rCEN | R | inferior parietal lobule | 2763 | 25.64 | 50 | -58 | 36 |
|  | R | middle frontal gyrus | 6180 | 23.21 | 44 | 38 | 26 |
|  | L | cerebellum | 912 | 18.00 | -32 | -62 | -32 |
|  | R | precuneus | 1239 | 15.04 | 2 | -32 | 40 |
|  | L | inferior parietal lobule | 810 | 13.71 | -52 | -48 | 44 |
|  | L | middle frontal gyrus | 1086 | 12.59 | -26 | 16 | 54 |
|  | R | middle temporal gyrus | 273 | 10.66 | 66 | -24 | -6 |
|  | R | posterior cingulate gyrus | 116 | 9.15 | 10 | -60 | 18 |
|  | L | caudate nucleus | 69 | 8.82 | -16 | 18 | 12 |
| latSM | R | precentral gyrus | 2383 | 26.40 | 50 | -4 | 30 |
|  | L | precentral gyrus | 2516 | 22.98 | -54 | -4 | 28 |
|  | R | cerebellum | 344 | 15.52 | 16 | -62 | -18 |
|  | L | cerebellum | 342 | 15.21 | -16 | -60 | -20 |
|  | L | precentral gyrus | 71 | 10.16 | -20 | -28 | 58 |
|  | R | precentral gyrus | 117 | 9.53 | 22 | -28 | 60 |
|  | Bi | supplementary motor area | 171 | 9.30 | 4 | 6 | 56 |
| supSM | Bi | supplementary motor area | 9090 | 30.57 | 2 | -26 | 54 |
|  | Bi | cerebellum | 481 | 11.06 | 10 | -42 | -24 |
|  | R | insular cortex | 137 | 9.90 | 36 | -22 | 16 |
|  | L | insular cortex | 188 | 9.71 | -34 | -22 | 16 |
| ifgFT | R | inferior frontal gyrus | 2999 | 28.66 | 46 | 20 | 24 |
|  | L | inferior frontal gyrus | 2329 | 23.43 | -46 | 28 | 20 |
|  | L | cerebellum | 290 | 16.28 | -14 | -74 | -32 |
|  | L | inferior parietal gyrus | 642 | 12.57 | -30 | -58 | 40 |
|  | R | cerebellum | 135 | 12.15 | 8 | -74 | -26 |
|  | R | inferior parietal gyrus | 462 | 11.31 | 36 | -52 | 44 |
|  | Bi | superior frontal gyrus | 556 | 11.14 | 0 | 48 | 34 |
|  | L | inferior temporal gyrus | 85 | 8.90 | -46 | -50 | -12 |
|  | Bi | anterior cingulate cortex | 110 | 8.47 | 2 | 6 | 28 |
|  | R | superior temporal gyrus | 58 | 7.43 | 62 | -44 | 12 |
| BG | R | putamen | 2951 | 24.84 | 24 | 8 | 2 |
|  | L | putamen | 3051 | 24.75 | -24 | 6 | -2 |

**Table S1:** *Peak activations of ICN spatial maps and associated anatomical regions. (k_E_ = Number of voxels in cluster; T_max_ = Maximum value of T-statistic in cluster; R = Right hemispheric; L = Left hemispheric; Bi = Bihemispheric)*

|  |  | **MMT** |  | **HT** |
| --- | --- | --- | --- | --- |
| **Session** | **Format** | **Theoretical training** | **Practical training** | **Theoretical training** |
| **1** | Video | Introduction to mindfulness | Mindful breathing A | Sleep |
| **2** | Audio | *(Practice only)* | Mindful breathing A | Chronic pain |
| **3** | Audio | *(Practice only)* | Mindful breathing A | Light exposition and health |
| **4** | Video | Arriving in presence | Mindful breathing B | Sleep disturbances |
| **5** | Audio | *(Practice only)* | Mindful breathing B | Body memory |
| **6** | Audio | *(Practice only)* | Mindful breathing B | Migraine |
| **7** | Video | Arriving in the body | Bodyscan A | Burnout |
| **8** | Audio | *(Practice only)* | Walking meditation | Nutritional supplements |
| **9** | Audio | *(Practice only)* | Bodyscan A | Social inequality and health |
| **10** | Video | Subjectivity of perception | Bodyscan B | Sore muscles / Vegan diet |
| **11** | Audio | *(Practice only)* | Walking meditation | Time perception |
| **12** | Audio | *(Practice only)* | Bodyscan B | Gender specific health experience |
| **13** | Video | Communicating mindfully | Mindful attention to body sensations | Vitamins |
| **14** | Audio | *(Practice only)* | Mindful attention to body sensations | Health impacts of dieting |
| **15** | Audio | *(Practice only)* | Mindful attention to body sensations | Aging |
| **16** | Video | Non-judgement | Mindful attention to body sensations | Sugar |
| **17** | Audio | *(Practice only)* | Mindful listening | Maintaining a diet |
| **18** | Audio | *(Practice only)* | Mindful listening | Self-deceit |
| **19** | Video | Dealing with stress | Mindfully approaching emotions | Raw foods |
| **20** | Audio | *(Practice only)* | Mindfully approaching emotions | Migration and health |
| **21** | Audio | *(Practice only)* | Mindfully approaching emotions | Epigenetics |
| **22** | Video | Turning towards instead of turning away | Turning towards instead of turning away | Sensible footwear |
| **23** | Audio | *(Practice only)* | Approaching unpleasant feelings | Obsessive-compulsive disorder |
| **24** | Audio | *(Practice only)* | Awareness of thinking | Self-efficacy |
| **25** | Video | Positive qualities | Loving kindness | Busting breakfast myths |
| **26** | Audio | *(Practice only)* | Loving kindness | Cardiovascular diseases |
| **27** | Audio | *(Practice only)* | Loving kindness | Hypnotherapy |
| **28** | Video | Decentring | Open monitoring | Staying active in the office |
| **29** | Audio | *(Practice only)* | Open monitoring | Negative empathy |
| **30** | Audio | *(Practice only)* | Silent meditation | Pain perception |
| **31** | Video | Reflecting the course | Silent meditation | Physical activity |

**Table S2:** *Contents and delivery scheme of MMT and HT. MMT included both theoretical and practical instructions, whereas HT was limited to delivering informative content. (MMT = Mindfulness Meditation Training; HT = Health Training)*
